# MERTK Interactions with SH2-Domain Proteins in the Retinal Pigment Epithelium

**DOI:** 10.1371/journal.pone.0053964

**Published:** 2013-02-04

**Authors:** Shameka J. Shelby, Karen Colwill, Sirano Dhe-Paganon, Tony Pawson, Debra A. Thompson

**Affiliations:** 1 Department of Biological Chemistry, University of Michigan Medical School, Ann Arbor, Michigan, United States of America; 2 Samuel Lunenfeld Research Institute, Mount Sinai Hospital, Toronto, Ontario, Canada; 3 Structural Genomics Consortium, University of Toronto, Toronto, Ontario, Canada; 4 Department of Physiology, University of Toronto, Toronto, Ontario, Canada; 5 Department of Medical Genetics, University of Toronto, Toronto, Ontario, Canada; 6 Department of Ophthalmology and Visual Sciences, University of Michigan Medical School, Ann Arbor, Michigan, United States of America; Center for Regenerative Therapies Dresden, Germany

## Abstract

The receptor tyrosine kinase MERTK plays an essential role in the phagocytic uptake of shed photoreceptor membranes by the retinal pigment epithelium (RPE). A fundamental aspect of signal transduction by receptor tyrosine kinases involves autophosphorylation of tyrosine residues that recruit Src-homology 2 (SH2)-domain proteins to the receptor intracellular domain. The goal of the current study was to evaluate the interactions of human MERTK with SH2-domain proteins present in the RPE. The MERTK intracellular domain was expressed as a 6xHis-fusion protein (6xHis-rMERTK_571–999_), purified and phosphorylated. Ni^2+^-NTA pull downs were performed using 6xHis-rMERTK_571–999_ in incubations with recombinant phosphotyrosine-recognition sequences expressed as GST-fusion proteins. In addition, pull downs of native SH2-domain proteins were performed using 6xHis-rMERTK_571–999_ and protein homogenates from rat RPE/choroid. For both recombinant and native proteins, western analysis detected MERTK interactions with GRB2, PIK3R1 (P85α), VAV3, and SRC. Immunohistochemical analysis localized each protein to mouse RPE. In cultured RPE-J cells incubated with rod outer segments (OS), siRNA knockdown of Grb2 had no effect on OS binding, but significantly reduced OS uptake. Pik3r1 localized to early phagosomes along with Rab5 and Eea1. Phosphorylation and activation of Src was detected downstream of phagocytosis and Mertk activation. These findings suggest that MERTK signaling in the RPE involves a cohort of SH2-domain proteins with the potential to regulate both cytoskeletal rearrangement and membrane movement. Identification of the SH2-domain signaling partners of MERTK is an important step toward further defining the mechanism of RPE phagocytosis that is central to the function and survival of the retina.

## Introduction

Vertebrate photoreceptor cells function under conditions of high metabolic demand and light exposure that result in chronic damage to lipid and protein components of their specialized outer segment (OS) membranes [1]. As a result, photoreceptor cells undergo a process of continual renewal involving the addition of new membranes at the base of the OS just above the connecting cilium, and shedding spent membranes from the OS tips [2]. The retinal pigment epithelium (RPE) removes the shed debris from the subretinal space by phagocytic uptake [3], a process that is critical for photoreceptor cell survival, as these cells depend on close association with the RPE for oxygen and nutrients, and for ionic homeostasis of the subretinal space [4]. A key insight into the molecular mechanism of phagocytic uptake by the RPE resulted from the discovery that inherited retinal degeneration in Royal College of Surgeons (RCS) dystrophic rats results from a deletion in the gene encoding Mertk [5]. As a consequence of this loss-of-function mutation, shed OS membranes can be bound but not taken up by the RPE [6]. Subsequent studies identified disease-associated mutations in the gene encoding human MERTK in individuals affected by a relatively rare form of juvenile-onset retinitis pigmentosa [7], [8], [9], [10], [11].

MERTK is a receptor tyrosine kinase belonging to the TAM receptor family (Tyro-3, Axl, Mer), whose ligands include the vitamin K-dependent proteins growth arrest-specific protein 6 (gas6) and protein S [12], as well as the retinal dystrophy-gene products Tulp1 and Tubby [13]. MERTK is expressed in monocytes and tissues of epithelial and reproductive origin [14] where it contributes to a number of cellular processes including cell proliferation and survival, down-modulation of pro-inflammatory signals, and clearance of apoptotic cells [15]. Studies of the mechanism of apoptotic cell clearance by transfected HEK-293T cells suggest that MERTK signaling involves cross-talk with αvβ5 integrin, resulting in activation of focal adhesion kinase (FAK) via SRC family non-receptor tyrosine kinases (SFKs) [16]. In both macrophages and the RPE, proteolytic cleavage of MERTK has been proposed to act in a negative feedback loop to limit phagocytic particle binding by αvβ5 integrin [16], [17]. In addition, MERTK has been shown to drive the redistribution of myosin II that is essential for the normal phagocytic function of the RPE, potentially by regulating the formation or closure of the phagocytic cup [18].

A central step in the MERTK signaling mechanism is the activation of receptor tyrosine kinase activity resulting in trans-autophosphorylation. Three tyrosine residues, Y-749, Y-753, and Y-754 present within the activation loop of the (human) MERTK-kinase domain have been identified as sites of autophosphorylation [19]. Receptor tyrosine phosphorylation serves to generate docking sites for signaling molecules, including Src-homology 2 (SH2) domain proteins that function as enzymes and adapter proteins [20]. Previous studies of MERTK-associated proteins in myeloid cells identified interactions with multiple SH2-domain proteins [21], [22], [23]. The fundamental role of SH2-domain proteins in MERTK-downstream signaling suggests that they play an essential role in the mechanism of RPE phagocytosis. In the present study, analysis of expression, protein interactions, and functional assays have been used to identify SH2-domain proteins in the RPE with the potential to signal downstream of MERTK and upstream of cellular remodeling. The findings suggest that MERTK interacts with multiple signaling partners in the RPE, including SH2-domain proteins that regulate cytoskeletal rearrangement and membrane movement in other professional phagocytes.

## Results

### Development of Study Tools and Focus

To identify potential MERTK-signaling partners in the RPE, expression analysis was coupled with a screening strategy that focused on candidate SH2-domain proteins selected from an extensive cDNA library encoding the corresponding phosphotyrosine-recognition sequences as GST-fusion proteins [20]. The recombinant human SH2-domains (rSH2-domains) were expressed in *E. coli* and purified by GSH-affinity and size-exclusion chromatography. Constructs encoding the full-length human MERTK cytoplasmic domain (rMERTK_571–999_), as well as a truncated construct (rMERTK_571–864_) [24], were expressed in *E. coli* as 6xHis fusion proteins and purified using Ni^2+^-NTA affinity and size-exclusion chromatography. To confirm autophosphorylation of tyrosine residues in the kinase domain, rMERTK_571–864_ was subjected to tryptic peptide analysis using MALDI mass spectrometry (MALDI-MS). Ion fragmentation patterns for three peptides designated P1, P2, and P3 identified three sites of tyrosine phosphorylation in the catalytic domain, Y749, Y753, and Y754, in agreement with previously published data [19] (Figure S1). To evaluate protein-protein interactions, Ni^2+^-NTA pull downs were performed using rMERTK_571–999_ (corresponding to the full-length cytoplasmic domain) with purified rSH2-domain fusion proteins, and also with rat RPE/choroid homogenates. Candidates for analysis included SH2-domain proteins previously implicated in MERTK downstream signaling. Expression in the RPE was evaluated at the transcript and protein level in cultured RPE-J cells, and in RCS congenic and dystrophic rats. The combined results led to a focus on protein families previously implicated in mechanisms of phagocytic uptake, including growth factor receptor-bound proteins (GRB), phosphatidylinositol 3-kinase regulatory subunit alpha (PIK3R1 or P85α), vav proto-oncogenes (VAVs), and SRC-family kinases (SFKs), as described in detail below.

### GRB2

**Figure 1:**
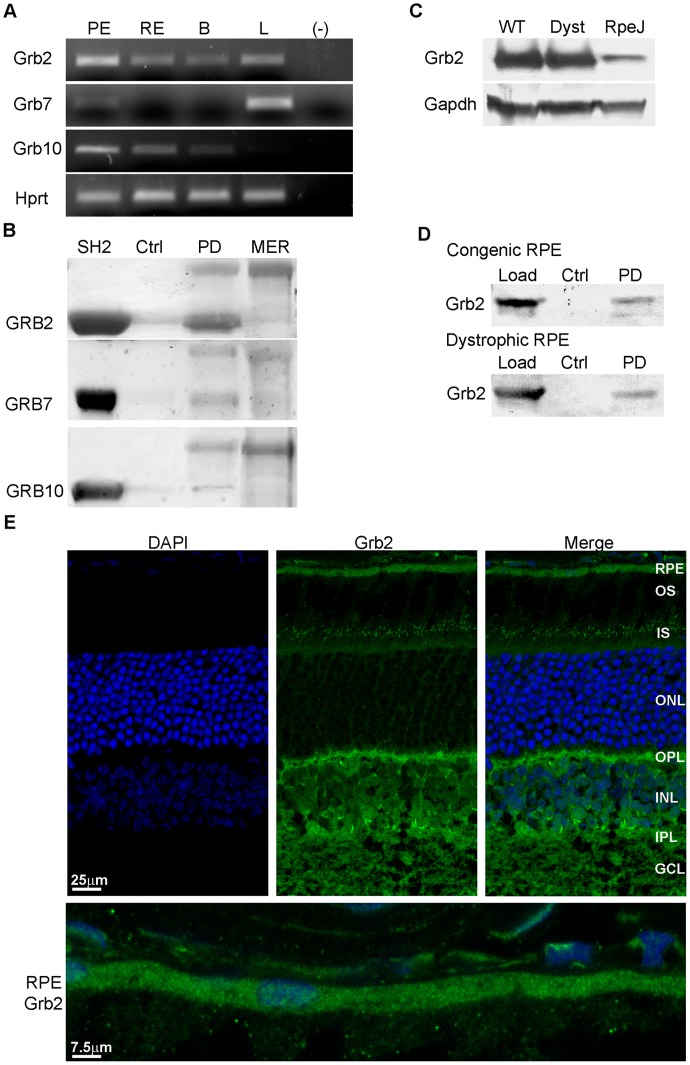
Grb2 is expressed and interacts with MERTK in the RPE. (A) *Grb* and *Hprt* transcripts amplified by RT-PCR from mouse RPE/choroid, retina, brain, and liver total RNA. Hprt primers served as control. PE, RPE/choroid; RE, retina; B, brain; L, liver; (-), no cDNA. (B) 6xHis-rMERTK_571–999_ and GRB GST-SH2-domains were purified from *E. coli* and their interactions evaluated using Ni^2+^-NTA pull downs. Coomassie blue-stained proteins of SDS gels are shown. SH2, GRB GST-SH2 domain loading control; Ctrl, negative control omitting rMERTK; PD, pull down of GRB SH2-domain and rMERTK_571–999_; MER, rMERTK_571–999_ loading control. (C) Grb protein immunoreactivity on western blots of RPE/choroid protein homogenates from 4 wk old RCS congenic and dystrophic rats at 2 h post light-onset, and of RPE-J cell homogenates, probed with antibodies recognizing Grb2. WT, congenic RCS-rat RPE/choroid; Dyst, dystrophic RCS-rat RPE/choroid; RpeJ, RPE-J cells. (D) Ni^2+^-NTA pull downs of endogenous proteins from RPE/choroid homogenates from RCS congenic and dystrophic rats incubated with or without 6xHis-rMERTK_571–999_. Western blots of the recovered proteins were probed with antibodies recognizing Grb2. Load, input RPE/choroid homogenate; Ctrl, Ni^2+^-NTA resin with homogenate alone; PD, pull down including rMERTK_571–999_ and homogenate. (E) Grb2 localization on retina/RPE/choroid cryosections from 4 wk old BALB/c mice imaged using indirect immunofluorescence with confocal microscopy. A selected area of RPE was enlarged to show specific localization to this cell layer. RPE, retinal pigment epithelium; OS, outer segments; IS, inner segments; ONL, outer nuclear layer; OPL, outer plexiform layer; INL, inner nuclear layer; IPL, inner plexiform layer; GCL, ganglion cell layer.

A key role of GRB proteins is their ability to function as adapters for GEF proteins involved in RAS activation of downstream kinases that regulate multiple signaling processes and biological activities, including NF-κB control of inflammation and regulation of motor proteins involved in cellular movement [25], [26]. Previous studies in hematopoietic cells identified MERTK interactions with GRB2 [21], pointing to the importance of defining the role of GRB proteins in RPE phagocytosis. In the current study, RT-PCR was used to evaluate expression in mouse RPE/choroid, retina, brain, and liver. Transcripts encoding Grb2 were seen at relatively high levels in RPE/choroid when compared to levels present in retina, brain, and liver (Figure 1A). In contrast, transcripts encoding the Grb7 isoform were low in RPE/choroid and high in liver, whereas transcripts encoding the Grb10 isoform were high in RPE/choroid and retina, and low in liver. Assays of potential MERTK interactions using Ni^2+^-NTA pull downs of 6xHis-rMERTK_571–999_ incubated with rSH2-domain fusion proteins showed strong recovery of the recombinant GRB2 protein (Figure 1B), which exceeded that of recombinant GRB7 and GRB10, suggesting specificity in the interactions of MERTK with GRB isoforms. Analysis of endogenous protein expression on western blots showed strong Grb2 immunoreactivity in RPE/choroid from RCS congenic and dystrophic rats [27], [28], with less seen in the rat RPE-J cell line [29] (Figure 1C). Pull downs of endogenous proteins from mouse RPE/choroid homogenates using 6xHis-rMERTK_571–999_ resulted in specific recovery of Grb2 seen on western blots (Figure 1D), and was not obtained using 6xHis-rMERTK_571–864_ missing the C-terminal sequence (data not shown). Immunohistochemical analysis of retina/RPE/choroid cryosections from BALB/c mice confirmed the presence substantial *Grb2* expression in both the RPE and neural retina, with marked immunoreactivity present in the interneuron and ganglion cell layers (Figure 1E), reflecting the fundamental role of Grb2 in a wide range of signaling processes. Taken together, these findings demonstrate the ability of the current approach to capture MERTK interactions with endogenous proteins, and are consistent with previous studies showing GRB2 binding to MERTK-Y867 [21].

### Grb2 in RPE Phagocytosis

**Figure 2:**
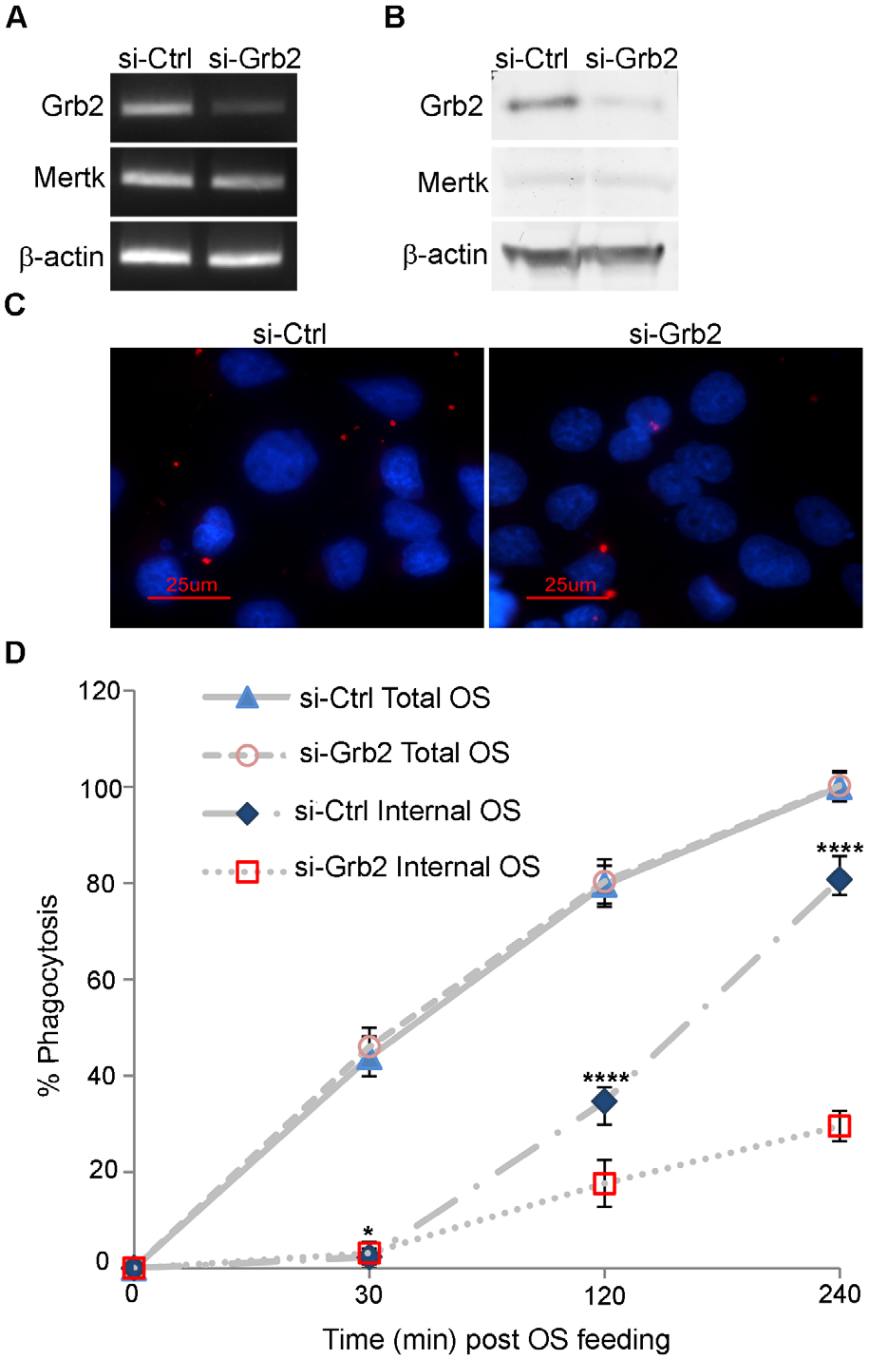
Grb2 silencing decreases phagocytic uptake in RPE cells. RPE-J cells were transfected with a pool of Grb2 targeting siRNAs or a non-targeting siRNA control, and expression was evaluated 5 days later. (A) Transcript levels for *Grb2*, *Mertk*, and *β-actin* evaluated using RT-PCR. (B) Protein levels of Grb2, Mertk, and β-actin evaluated by western blot analysis of RPE-J cell homogenates. (C-D) Phagocytic activity assays. (C) Confocal images of representative fields of RPE-J cells showing ingested OS labeled in red, and DAPI-stained nuclei shown in blue. Cells were incubated with AlexaFluor 555-labeled bovine rod OS for 4 h and quenched by addition of trypan blue. (D) OS ingestion and binding were quantified for three independent assays and plotted as a percentage of control total OS after 4 h that was set as 100%. Error bars represent mean ± SEM, n = 3. P values were calculated using Student’s *t* test, and are as shown: p**<0.05, p****<0.00005.

To evaluate the effect of Grb2 loss-of-function on RPE phagocytosis, siRNAs were used to deplete *Grb2* transcripts in sub-confluent rat RPE-J cells, followed by quantitative assays of OS binding and uptake. Transient transfection of RPE-J cells with a pool of four Grb2 targeting siRNAs resulted in efficient knockdown at both the transcript and protein level (Figures 2A, 2B). In contrast, *Grb2* expression was retained in cells transfected with a control non-targeting siRNA. Equivalent levels of Mertk and β-actin were present in both treated and control cells. Furthermore, phalloidin staining of the actin cytoskeleton, and immunostaining with antibodies against the ZO-1 protein present in tight junctions showed little disruption of cellular integrity (Figure S2A). In assays of phagocytic uptake using bovine OS labeled with AlexaFluor 555, RPE-J cells treated with *Grb2* targeting siRNAs exhibited significantly less activity (∼60% at the 6 h time point) than cells treated with control non-targeting siRNA (p<0.0005) (Figures 2C, 2D). In both cases, OS binding was unaffected (p>0.05). Taken together, these findings support the view that the interaction of endogenous Grb2 with MERTK contributes, directly or indirectly, to the phagocytic activity of the RPE.

### PIK3R1 (P85α)

**Figure 3:**
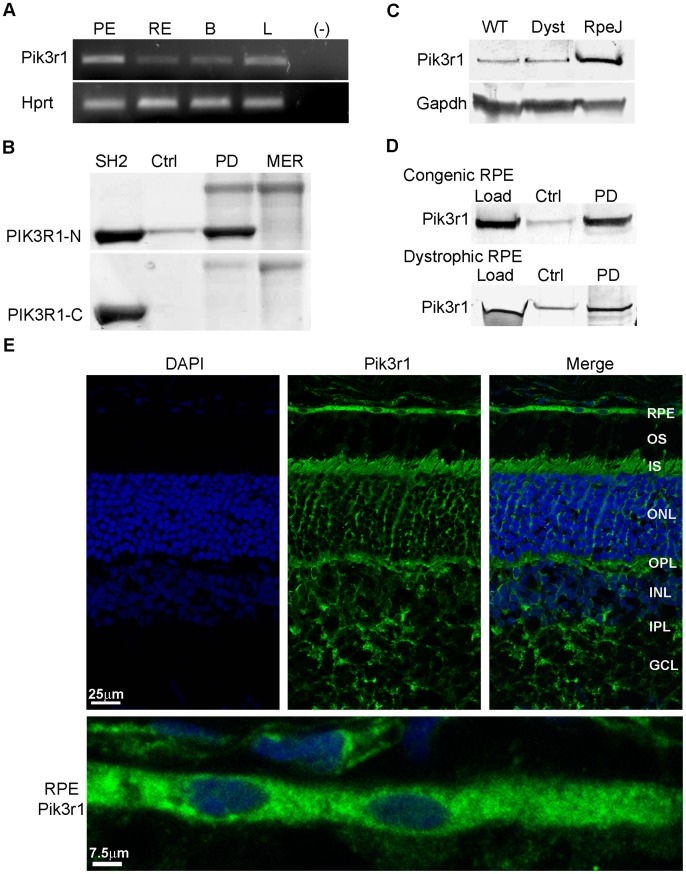
Pik3r1 is expressed and interacts with MERTK in the RPE. (A) *Pik3r1* and *Hprt* transcripts amplified from mouse tissues by RT-PCR. (B) Ni^2+^-NTA pull downs of recombinant PIK3R1 GST-SH2 domains incubated with or without 6xHis-rMERTK_571–999_ and evaluated on SDS-gels. (C) Pik3r1 immunoreactivity on western blots of rat RPE/choroid and RPE-J cell homogenates. (D) Ni^2+^-NTA pull downs with RPE/choroid protein homogenates from RCS congenic and dystrophic rats incubated with or without 6xHis-rMERTK_571–999_, and evaluated by western analysis with antibodies recognizing Pik3r1. (E) Pik3r1 localization on cryosections of mouse retina/RPE/choroid. Details as in Figure 1.

Stimulation of phosphoinositide*-*3-kinase (PI3K) activity by activated receptor tyrosine kinases plays a central role in downstream signaling mechanisms, including those involved in regulating inflammatory responses [30], [31]. Analysis of the expression of the PI3K regulatory subunit Pik3r1 in mouse tissues showed that transcript levels were relatively high in RPE/choroid compared to levels in retina and brain (Figure 3A). Ni^2+^-NTA pull downs with 6xHis-rMERTK_571–999_ showed specific recovery of a PIK3R1-fusion protein corresponding to the SH2-domain present in the amino-terminal half of the regulatory subunit (PIK3R1-N), which was not seen with a fusion protein corresponding to the SH2-domain present in the carboxy-terminal half (PIK3R1-C) (Figure 3B). Western analysis showed equivalent levels of Pik3r1 protein in RPE/choroid from congenic and dystrophic rats, and higher levels in RPE-J cells (Figure 3C). Pull downs of 6xHis-rMERTK_571–999_ incubated with RPE/choroid homogenates showed specific recovery of the endogenous Pik3r1 protein from both congenic and dystrophic samples (Figure 3D). Immunohistochemistry on mouse retina/RPE/choroid cryosections showed strong Pik3r1 immunoreactivity in the RPE and photoreceptor inner segments, and less intense labeling present in the inner retina (Figure 3E). Taken together, these results are the first to demonstrate a direct interaction of MERTK with PI3K *in vitro* and with native RPE protein.

### Pik3r1 in the Early Phagosome

**Figure 4:**
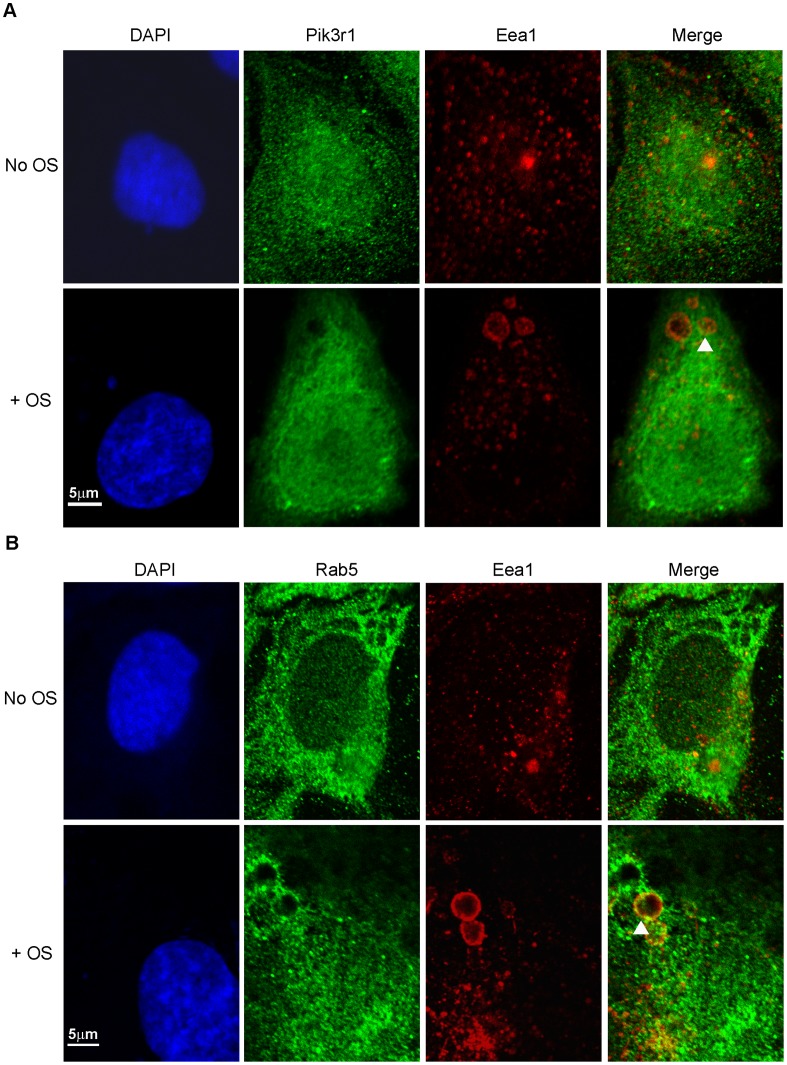
Pik3r1 co-localizes to early phagosomes with Eea1 and Rab5 during OS uptake. RPE-J cells were incubated with, or without, isolated bovine OS for 4 h. Cells were fixed and stained with antibodies recognizing Pik3r1 and Eea1 with AlexaFluor 488 secondary (A), or recognizing Rab5 and Eea1 with AlexaFluor 555 secondary (B), and viewed using fluorescence confocal microscopy. Areas showing co-localization appear as yellow in the merged images and are marked by white arrowheads.

Previous studies have shown that PI3K participates in early events necessary for phagosome formation and maturation [32]. To investigate the role of Pik3r1 relative to phagosome formation in the RPE, cultured RPE-J cells were incubated with or without bovine rod OS, and indirect immunofluorescence microscopy was performed using antibodies against Pik3r1, as well as two markers for phagosome formation: early endosomal antigen 1 (Eea1) and Rab5 GTPase [31]. In RPE-J cells incubated with OS, each of these three proteins were found to co-localize with internalized rhodopsin-containing structures likely corresponding to early phagosomes (Figure S3). In cells incubated without added OS, Pik3r1 labeling appeared uniform and diffuse (Figure 4A), whereas Eea1 labeling was associated with small discrete vesicles present throughout the cytoplasm (Figure 4A), and Rab5 labeling was widespread and punctate (Figure 4B). In contrast, in RPE-J cells incubated with rod OS, Pik3r1 and Eea1 labeling was seen to co-localize on large vesicular structures (Figure 4A). These structures were similar in appearance to those exhibiting co-localization of Eea1 and Rab5 (Figure 4B). These findings are consistent with localization of Pik3r1 to OS-containing phagosomes in the RPE, and place it in proximity to Rab5, an established participant in the formation of endosomes and phagosomes [33]. Taken together, ability of PIK3R1 to undergo direct interaction with MERTK, coupled with its colocalization with early endosome markers EEA1 and RAB5, as well as its potential to act as an effector of RAB5 [33], suggest that PIK3R1 activation downstream of MERTK signaling may represent a novel mechanism contributing to phagosome formation in the RPE.

### VAV Proteins

**Figure 5:**
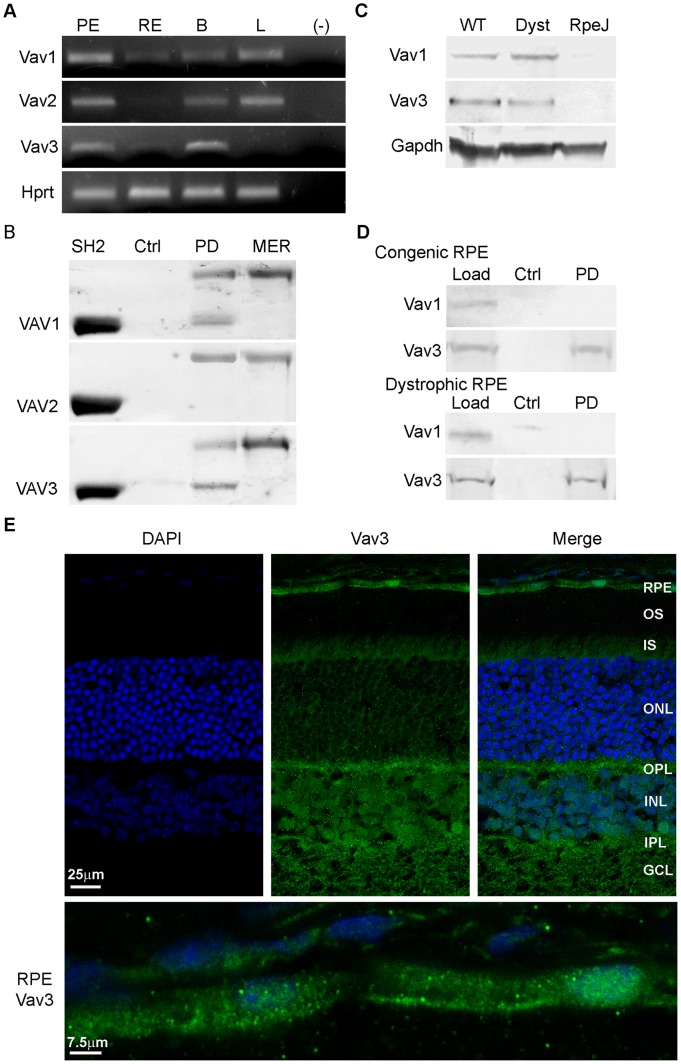
Vav3 is expressed and interacts with MERTK in the RPE. (A) *Vav* and *Hprt* transcripts amplified from mouse RPE/choroid by RT-PCR. (B) Ni^2+^-NTA pull downs of recombinant VAV GST-SH2 domains incubated with or without 6xHis-rMERTK_571–999_ and evaluated on SDS-gels. (C) Vav protein immunoreactivity on western blots of rat RPE/choroid and RPE-J cell homogenates. (D) Ni^2+^-NTA pull downs with RPE/choroid protein homogenates from RCS congenic and dystrophic rats incubated with or without 6xHis-rMERTK_571–999_, and evaluated by western analysis with antibodies recognizing Vav1 and Vav3. (E) Vav3 localization on cryosections of mouse retina/RPE/choroid. Details as in Figure 1.

The VAV family of proteins, VAV1, VAV2, and VAV3 serve as guanine nucleotide exchange factors (GEFs) for RAC1 GTPase that contributes to cytoskeletal rearrangement through effects on actin polymerization [34]. Previous studies reported that VAV1 undergoes interaction with MERTK independent of receptor phosphorylation status [23]. In the current study, analysis of expression in mouse RPE/choroid showed transcripts encoding all three VAV isoforms (Figure 5A). Pull-down assays with 6xHis-rMERTK_571–999_ showed interaction with rSH2-domain fusion proteins corresponding to VAV1 and VAV3, but not VAV2 (Figure 5B). Western analysis showed both Vav1 and Vav3 were present in protein homogenates of RPE/choroid from congenic and dystrophic rats, but not RPE-J cells (Figure 5C). Pull downs from RPE/choroid homogenates showed interaction of 6xHis-rMERTK_571–999_ with endogenous Vav3, but not with Vav1 (Figure 5D). Immunohistochemical analysis of retina/RPE/choroid cryosections showed punctate localization of Vav3 in the RPE, and diffuse labeling in the inner retina (Figure 5E). Viewed together, these findings suggest that the association of VAV3 comprises the predominant MERTK interaction occurring with VAV family members in the RPE.

### SRC

**Figure 6:**
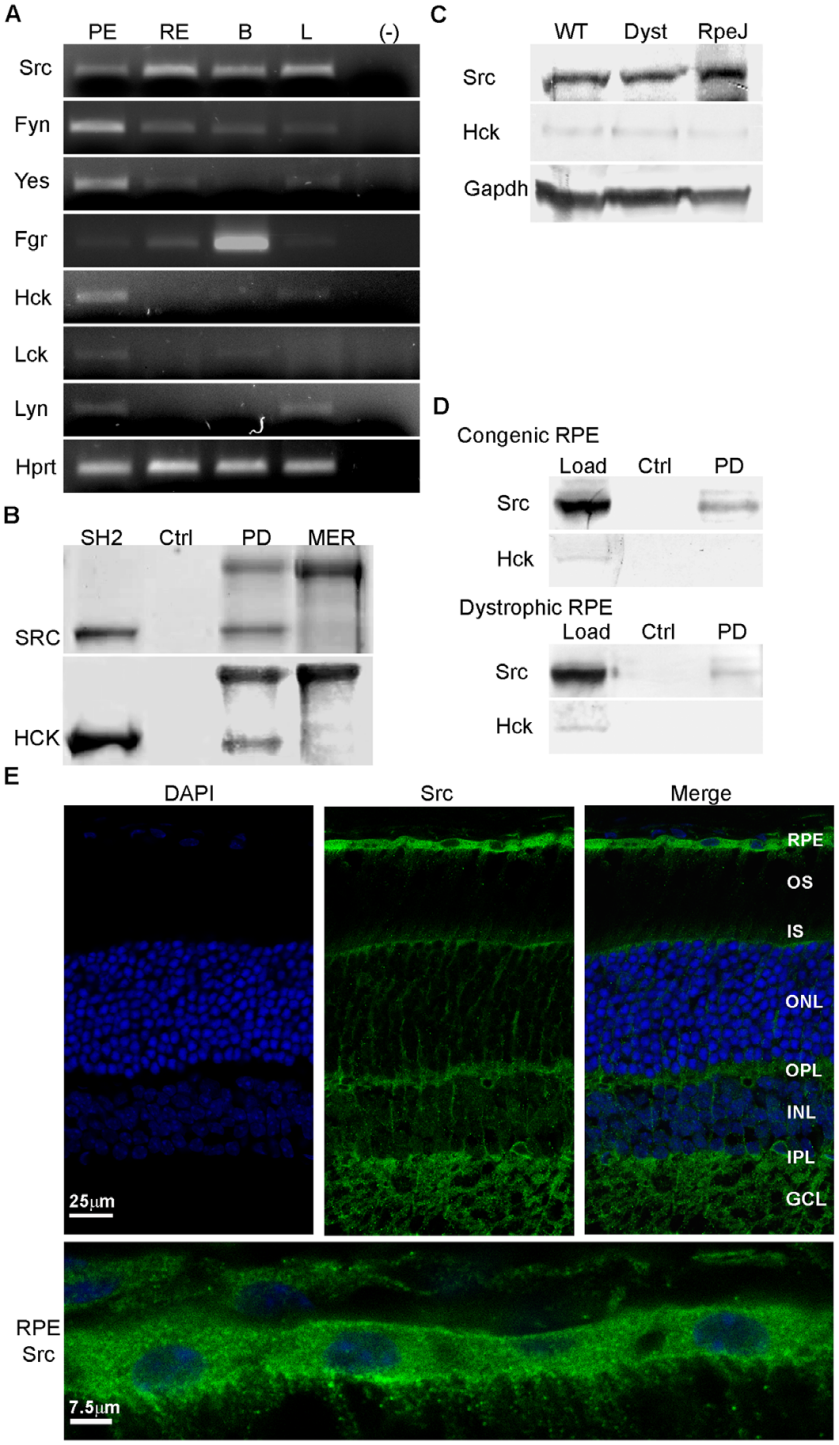
Src is expressed and interacts with MERTK in the RPE. (A) Src family kinase and *Hprt* transcripts amplified from mouse RPE/choroid by RT-PCR. (B) Ni^2+^-NTA pull downs of recombinant SRC and HCK GST-SH2 domains incubated with or without 6xHis-rMERTK_571–999_ and evaluated on SDS-gels. (C) Src and Hck immunoreactivity on western blots of rat RPE/choroid and RPE-J cell homogenates. (D) Ni^2+^-NTA pull downs with RPE/choroid protein homogenates from RCS congenic and dystrophic rats incubated with or without 6xHis-rMERTK_571–999_, and evaluated by western analysis with antibodies recognizing Src and Hck. (E) Src localization on cryosections of mouse retina/RPE/choroid. Details as in Figure 1.

SRC-family kinases (SFKs) are intracellular tyrosine kinases that act downstream of receptor activation to powerfully impact signaling. SFKs have been shown to play a role in apoptotic cell clearance by dendritic cells [22] and to contribute to immunoreceptor signaling activated upon particle uptake [35]. In the current study, analysis of expression in mouse RPE/choroid identified transcripts encoding nearly the entire SRC family, including *Src*, *Fyn*, *Fgr*, *Yes*, *Hck*, *Lyn*, and *Lck* (Figure 6A). Transcripts encoding *Hck*, *Fyn*, and *Yes* appeared to be relatively more abundant in RPE/choroid versus retina. Pull-down assays with 6xHis-rMERTK_571–999_ showed specific interaction with the rSH2-domain fusion proteins corresponding to SRC and HCK (Figure 6B). Western analysis showed that Src was present in the RPE/choroid from congenic and dystrophic rats, and RPE-J cells, while Hck protein levels were significantly lower in each case (Figure 6C). However, rMERTK_571–999_ pull downs with RPE/choroid homogenates from congenic and dystrophic rats showed interaction with endogenous Src, but interaction with Hck was not detected (Figure 6D). Immunohistochemical analysis of Src showed strong labeling throughout the RPE, as well as the inner retinal layer (Figure 6E). In the RPE, significant labeling extended toward the apical microvilli. These results suggest that SRC is a candidate for direct interaction with MERTK and the regulation of downstream effects on RPE function.

### SRC Activation Downstream of MERTK

**Figure 7:**
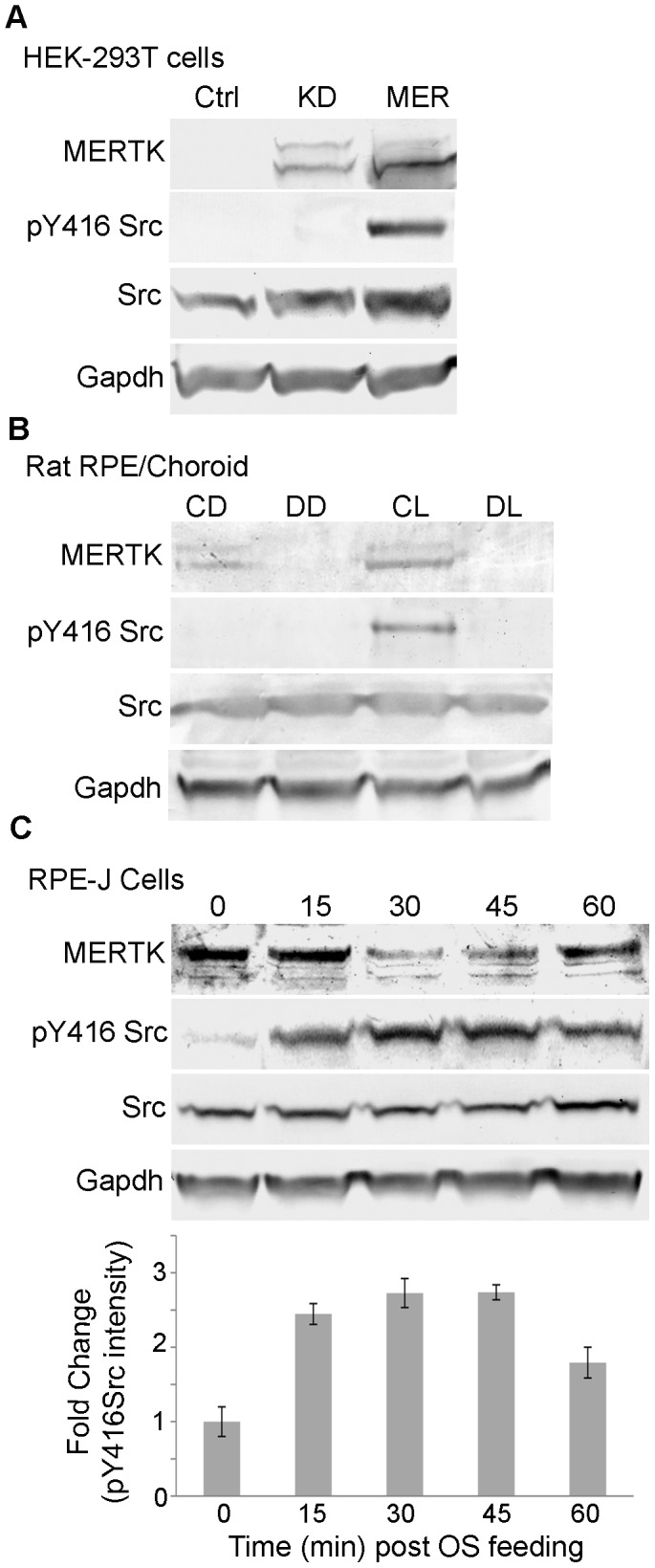
Src is phosphorylated on Y416 in response to MERTK activation in the RPE. Immunoreactivity of antibodies recognizing pY416 Src, Src, Mertk (C-terminal antibody), and Gapdh on western blots of cell and tissue homogenates. (A) HEK-293T cells transfected with full length MERTK (Mer) or kinase-dead R844C-MERTK (MerKD). (B) RPE/choroid homogenates from 4 wk old RCS congenic and dystrophic rats harvested at 1 h prior to light onset, and 1.5 h post light onset (peak of phagocytic uptake). CD, congenic rat RPE/choroid in the dark; DD, dystrophic in the dark; CL, congenic at peak activity; DL, dystrophic taken at peak activity. (C) RPE-J cells were incubated with OS and lysed at the times indicated, followed by western blot analysis. Results for pY416 Src are plotted as the mean fold change in band intensity. Error bars represent mean ± SEM, n = 3.

Previous studies of apoptotic cell clearance have proposed that SFKs facilitate crosstalk between MERTK and αvβ5 integrin in downstream signaling to RAC1 involved in cytoskeletal reorganization [16], [29], [36], however, the mechanistic details and the specific SFK(s) involved have not been elucidated. In the current study, the activation status of SRC was evaluated using an antibody that recognizes the active form of the protein that is phosphorylated on tyrosine residue 416 (pY416). This analysis showed formation of pY416 SRC in HEK-293T cells transfected with active full-length MERTK, but not with kinase-dead MERTK (Figure 7A). In addition, formation of pY416 Src was seen in the RPE/choroid of congenic RCS rats sacrificed during peak phagocytic uptake, but was not seen in animals sacrificed before light onset, or in dystrophic RCS rats sacrificed at either time (Figure 7B). Formation of pY416 Src was also seen in RPE-J cells incubated with rod OS, appearing highest at 30 and 45 min post feeding (Figure 7C). Viewed together, these findings suggest that SRC activation in the RPE can result from direct interaction with activated MERTK, and leave open the possibility that additional SFKs may function downstream of MERTK signaling in phagocytosis.

## Discussion

Phagocytic uptake by the RPE requires multiple activities that regulate cellular plasticity, including cytoskeletal reorganization and membrane remodeling, and also contribute to down-modulating pro-inflammatory responses. Although not required for binding of effete membranes, MERTK signaling is essential for initiating phagocytic uptake. The current studies show that MERTK interactions in the RPE/choroid and other phagocytic cell types involve an overlapping set of core SH2-domain proteins that are not down regulated in response to MERTK loss-of-function in the RCS rat. These findings lead to a new appreciation of the phagocytic mechanism adapted to the highly-specialized function of the RPE.

Changes in actin polymerization needed for cytoskeletal reorganization are regulated by members of the RHO family of GTPases, including the small G-proteins RAC1, CDC42, and RHO A [37]. Activation of RHO family GTPases requires interaction with guanine nucleotide exchange factors (GEFs) necessary for GDP/GTP exchange [34]. These include the VAV family of SH2-domain proteins that act as GEFs for RAC1 involved in regulating F-actin recruitment. Previous studies in macrophages showed that VAV1 interacts with MERTK independent of its phosphorylation status [23]. In the current studies, both VAV1 and VAV3 were shown to interact with MERTK as recombinant proteins, but only native Vav3 was found to interact in assays of endogenous rat RPE proteins. Vav3 was also the major isoform seen in the mouse RPE using immunohistochemical analysis. In contrast, expression of VAV family proteins was not detected in cultured RPE-J cells that retain the capacity to perform MERTK-mediated phagocytic uptake. In addition, previous studies of Vav2/3 knockout mice have shown that Vav loss-of-function results in a glaucoma phenotype, but not retinal degenerative disease [38]. Taken together, these findings are consistent with the view that direct activation of VAV proteins by MERTK is not essential for the uptake phase of phagocytosis, but likely fulfills another signaling role downstream of MERTK activation in the RPE.

Recent studies have shown that RAC1 activation occurs downstream of αvβ5 integrin, but does not require MERTK, suggesting that integrin signaling through the CRKII/p130CAS/DOCK180/ELMO complex is the primary mechanism involved in activating RAC1 in the RPE [36]. A model of MERTK mediated phagocytosis based on these studies invokes the involvement of SFKs in crosstalk between MERTK and αvβ5 integrin in regulating downstream signaling to RAC1 [16], [29], [36], [39]. Crosstalk between integrins, RHO-family GTPases, and SFKs is well documented in signaling involved in cellular adhesion [40]. The current studies establish that MERTK interacts with recombinant SRC, as well as with endogenous Src in the rat RPE. In addition, activation of MERTK promotes Src activation as evidenced by phosphorylation of Y416. Transcripts encoding the known mammalian SFKs, with the exception of Blk, were detected in mouse RPE/choroid. This suggests there are multiple opportunities for SFKs to directly participate in MERTK signaling in the RPE, as well as in crosstalk with integrins involved in cytoskeletal reorganization. Such mechanisms have the potential to make significant contributions to cellular motility, in that SFKs are important in regulating other RHO family GTPases, including RHOA which functions to induce actomyosin contractility [41].

Myosin motor proteins responsible for actin motility are an important driving force in phagosome formation [42]. In the RPE, myosin II has been shown to interact with MERTK and contribute to phagosome formation [18], whereas myosin VIIa has been implicated in phagosome trafficking [43]. Previous studies of MERTK-associated proteins in hematopoietic cells identified the adapter protein GRB2 [21] and established that this interaction is a prerequisite for phagocytic clearance of apoptotic cells [25]. In addition, GRB2 signaling to myosin and dynamin via the RAS pathway has been shown to induce membrane ruffling and cell migration [44]. The current studies show that MERTK interacts with endogenous Grb2 present in the RPE. Consistent with previous findings that Y867 is required for the interaction of recombinant MERTK and GRB2 [21], native Grb2 was found to interact with the full-length rMERTK_571–999_ intracellular domain, but not with rMERTK_571–864_. In addition, knockdown of Grb2 was found to inhibit the phagocytic uptake of ROS by RPE-J cells in culture, although it was not established whether this was due to a direct effect on the phagocytic mechanism or other aspects of cell function and viability. Taken together, these findings point to a conserved role for GRB2 in apoptotic cell clearance, endocytosis, and RPE phagocytosis.

In phagocytic uptake by macrophages, formation of the phagocytic cup as well as phagosome maturation require the involvement of both class I and III PI3Ks [32]. In Fc-receptor signaling, one role of PI3K is to generate the phosphoinositides that recruit RAB5 GTPase and early-endosomal markers (including EEA1) to the phagosome [30], [31], [33]. Previous studies using the PI3K inhibitors wortmannin and LY294002 have shown that loss of PI3K activity inhibits RPE phagocytosis [45]. In hematopoetic cells, the PIK3R1 regulatory subunit of class I PI3K has been shown to interact indirectly via GRB2 with a FMS-MERTK chimera [21]. In the current studies, recombinant MERTK was shown to interact with endogenous Pik3r1, but the possibility that endogenous Grb2 contributed to this interaction cannot be excluded. However, studies using purified recombinant proteins also showed that PIK3R1 interacts with the rMERTK_571–999_ intracellular domain. The site of this interaction, as well as those of VAV3 and SRC, are not predicted using various algorithms [46] and remain to be experimentally determined. Taken together, these findings suggest that PI3K can undergo both direct and indirect interactions with MERTK. The finding that Pik3r1 colocalizes with both Eea1 and Rab5 GTPase in phagosomes formed during uptake of OS by RPE-J cells suggests that this mechanism is conserved among professional phagocytes.

In immune cells, an important role of MERTK is to dampen pro-inflammatory responses resulting from apoptotic cell binding [47]. This is accomplished in part by GRB2 and PI3K signaling downstream of autophosphorylation of MERTK-Y867 that contributes to down regulation of NF-κB [21], [25]. Studies in dendritic cells also have shown that SRC signaling downstream of MERTK plays an important role in immunomodulation [22]. The finding that GRB2, PI3K, and SRC likely contribute to MERTK signaling in the RPE raises the important possibility that this mechanism plays a critical role in controlling inflammatory responses elicited by OS uptake, as well as those associated with aging and disease.

Establishing the identities of MERTK-interacting SH2-domain proteins in the RPE provides new insight into downstream signaling potentially linked to various aspects of the phagocytic mechanism. The role of MERTK loss-of-function in inherited retinal degeneration suggests that key signaling partners may be candidates for involvement in this group of diseases. Thus, increased knowledge of the phagocytic mechanism has the potential to advance our understanding of the causes of RPE dysfunction occurring in aging and disease, as well as further efforts to develop targeted therapeutic strategies to improve RPE function.

## Materials and Methods

### Animals

Experimental procedures involving animals were performed in accordance with the guidelines and under the approval of the University Committee on Use and Care of Animals at the University of Michigan, and were in compliance with the statement for ethical care and use of animals of the Association for Research in Vision and Ophthalmology (ARVO). C57BL/6J and BALB/C mice were bred from animals obtained from the Jackson Laboratories. Pigmented dystrophic (RCS-*p*+) and non-dystrophic (RCS-*rdy*+*p*+) rats were bred from the strains described in [27], [28]. Mice and rats were housed in a 12-h/12-h light-dark cycle (∼300 lux room light) and were euthanized by CO_2_ inhalation approximately 2 h after light onset, a time when levels of RPE phagocytic uptake are near maximal [48].

### Materials

Primary antibodies: GRB2, Santa Cruz Biotechnology; PIK3R1, VAV1, VAV3, and HCK, Millipore; c-SRC, pY416 SRC, and EEA1, Cell Signaling Technology; GAPDH, Ambion; β-actin and RAB5, Abcam. AlexaFluor 555, AlexaFluor 488-conjugated anti-rabbit IgG, AlexaFluor 555-conjugated anti-mouse IgG, and Sybr safe were from Invitrogen. Complete protease inhibitors, PhosSTOP phosphatase inhibitors, Fugene transfection reagent, and AmpliTaq Gold polymerase were from Roche Diagnostics. Ni^2+^-NTA resin, RNeasy kit, Superscript II, and oligo-dT were from Qiagen. RPE-J and HEK-293T cells were from ATCC. Other chemicals and reagents were from Sigma. The pcDNA 3.1+ expression vectors encoding full-length MERTK and kinase-dead R844C-MERTK have been previously described [8].

### RT-PCR Analysis of SH2-domain Protein Expression

Total RNA was isolated from freshly-dissected RPE/choroid, retina, liver, and brain from C57BL/6 mice using RNAeasy kits, and first-strand cDNAs were generated using Superscript II and oligo-dT. Sequences encoding SH2-domain proteins of interest were amplified with gene-specific primers flanking at least one intron in the genomic sequence (Table S1) using AmpliTaq Gold polymerase and the following cycling conditions: 1 cycle at 95°C for 10 min followed by 28 cycles at 95°C for 2 min, 60°C for 45 sec, 72°C for 2 min. Primers for hypoxanthine-guanine phosphoribosyltransferase (Hprt) served as a control. PCR products were analyzed by electrophoresis on agarose gels stained with Sybr safe. Comparisons of the relative levels of different gene products were made using the same RT reaction in amplifications containing a single pair of primers.

### Western Analysis of SH2-domain Protein Expression

Dissected samples of RPE/choroid from dystrophic and congenic RCS rats were harvested at times corresponding to peak phagocytic uptake [48], and confluent cultures of the rat RPE-J cell line that retains Mertk expression [29] were harvested after 3 days in culture. Tissues and cells were homogenized in 20 mM MOPS, 2 mM EGTA, 5 mM EDTA, 1% Triton X-100, 1 mM DTT, protease and phosphatase inhibitors; cellular debris was removed by low speed centrifugation; and protein concentrations of the supernates were determined using a modification of the Lowry method [49]. Protein samples (7.5 µg) were dissociated in 1× SDS sample buffer, electrophoresed on 10% polyacrylamide gels, and transferred to nitrocellulose. Blots were blocked and incubated with primary antibodies specific for SH2-domain proteins of interest, then with alkaline phosphatase-conjugated secondary antibodies, and developed using 5-bromo-4-chloro-3′-indolyphosphate p-toluidine and nitro-blue tetrazolium chloride.

### Immunohistochemical Analysis

Eyes from BALB/c mice perfused with 4% paraformaldehyde were washed with PBS, transitioned to sucrose/OCT, and flash frozen. Retinal cross sections (10 µm) were washed with PBS and permeabilized with PBS-T (0.125% Triton X-100 ); blocked with 1% bovine serum albumin, 10% normal goat serum, and 0.125% Triton X-100; and incubated with primary antibodies for 2 h, then with fluorophore-conjugated secondary antibodies for 1 h. Species and dilutions of primary antibodies were as follows: Rabbit anti-GRB2 (1∶300), anti-PIK3R1 (1∶300), anti-VAV3 (1∶300), anti-SRC (1∶300), anti-RAB5 (1∶300); Mouse anti-EEA1 (1∶300); AlexaFluor 488 anti-rabbit IgG (1∶500); AlexaFluor 555 anti-mouse IgG. Images were obtained by confocal fluorescence microscopy (Leica SP5). For immunohistochemistry with RPE-J cells, the cultures were challenged with isolated bovine rod OS [50], washed 3 times with PBS, and fixed with 4% paraformaldehyde for 30 min at room temperature. The cells were then processed using the same methods as for retinal cross sections.

### rSH2-domain Protein Expression and Purification

GST-tagged constructs in pGEX2T vectors encoding the phosphotyrosine-recognition sequences of SH2-domain proteins were previously generated from a library representing nearly the complete set of known SH2-domain proteins [51]. The constructs were transformed into BL21 DE3 Gold bacteria and large scale cultures were grown in Terrific Broth with glycerol plus ampicillin at 37°C to an OD_600_ of 0.8. Isopropyl β-D-1-thiogalactopyranoside (final concentration 0.1 mM) was added and the cells were incubated at 15°C overnight. The cells were pelleted and resuspended in phosphate buffered saline and lysed by French press. Glutathione-agarose beads were incubated with cleared lysates for 1 h at 4°C, washed with 10 volumes of PBS- 1% TritonX100, and eluted with buffer containing 8 mM glutathione, 50 mM Tris-HCl, pH 9.5. Fractions were collected and analyzed on SDS gels, and fractions containing rSH2-domains were pooled, concentrated to 1 mL, and loaded on a Sephacryl S-200 HR column (21×1 in). Fractions were collected at a rate of 0.5 mL/min, analyzed on SDS gels, and those containing purified rSH2-domains were pooled and concentrated.

### rMERTK Expression and Purification

Two His-tagged expression constructs encoding the human MERTK cytoplasmic domain, amino acid residues 571 to 864 (6xHis-rMERTK_571–864_) [23] and 571 to 999 (6xHis-rMERTK_571–999_), in the pET28a-LIC vector were amplified in bacterial cells as described above for rSH2-domains, with kanamycin replacing ampicillin in the cultures. Cells were pelleted and resuspended in lysis buffer containing 50 mM Tris-HCl, 500 mM NaCl, 5% glycerol, 1 mM β-mercaptoethanol, 2 mM imidazole, and 200 µM phenylmethylsulfonyl fluoride (PMSF) at pH 8, and lysed by French press. Ni^2+^-NTA resin was incubated with cleared supernatants with shaking for 1 h at 4°C, washed with 10 volumes of 10 mM imidazole in lysis buffer, and eluted with 200 mM imidazole in lysis buffer. The eluate was concentrated to 1 mL, chromatographed on Sephacryl S-200 HR as described above, evaluated on SDS gels, pooled, and concentrated. Recombinant MERTK was autophosphorylated by incubating with 10 mM ATP, 10 mM MgCl_2_ in gel filtration buffer at room temperature for 3 h and was stored at −80°C.

### Phosphotyrosine Analysis by MALDI-MS

Purified, phosphorylated 6xHis-rMERTK_571–864_ was digested by addition of porcine trypsin in 50 mM ammonium bicarbonate, 0.05% SDS, and incubated overnight at 37°C. The digested peptides were subjected to TiO_2_ selection to enrich for phosphorylated peptides and evaporated to dryness in a SpeedVac. The sample was dissolved in 5 µL 60% Acetonitrile and 0.1% Trifluoroacetic acid. 1 µL of sample was spotted on MALDI target plates and peptides were separated by liquid chromatography. Phosphopeptide analysis of the separated peptides was performed using a 4700 MALDI TOF/TOF mass spectrometer (Applied Biosystems) with peptide mass analysis using UniProt by the University of Michigan Protein Core Facility.

### rMERTK Pull Downs of rSH2-domains and Native SH2-domain Proteins

Purified GST-tagged-rSH2-domain proteins (10 µg) were incubated with 6xHis-rMERTK_571–999_ (10 µg) in 50 mM NaH_2_PO_4_, 300 mM NaCl, 10 mM imidazole, and 0.1 µM PMSF for 1 h at 4°C. 50 µL of Ni^2+^-NTA resin slurry was added and the incubation was continued for an additional 0.5 h. The beads were collected by brief centrifugation, washed three times in binding buffer including 50 mM imidazole, and eluted with binding buffer containing 500 mM imidazole. Negative controls omitted the 6xHis-tagged-rMERTK_571–999_. For native tissue, protein homogenates from RPE/choroid were obtained as described for western analysis. Ni^2+^-NTA pull downs of the protein homogenates with 6xHis-tagged-rMERTK_571–999_ (10 µg) were performed as described above.

### Cell Culture and Transfections

HEK-293T cells were maintained in DMEM supplemented with 10% FBS, 1 mM sodium pyruvate, and 1 mM penicillin/streptomycin at 37°C in 5% CO_2_. HEK-293T cells were transiently transfected with full-length MERTK and kinase-dead R844C-MERTK using FuGENE as recommended (Roche). Rat RPE-J cells were maintained in Dulbecco’s modified Eagle’s medium (DMEM) supplemented with 4% fetal bovine serum (FBS), and 1 mM non-essential amino acids at 33°C in 5% CO_2_. Rat Grb2 siRNAs were obtained as a Smartpool (Thermo Scientific) containing mixtures of four different duplexes to minimize silencing of unintended targets. ON-TARGET plus non-targeting siRNA (at the same concentration as the total pool of targeting siRNAs) served as a negative control. RPE-J cells (32,000 cells per well) were passaged into eight-well chamber slides, and 24 h later each well was transfected with 0.5 µg of the siRNAs plus 3.75 µL of DharmaFect 3 transfection reagent as recommended (Dharmacon). The cells were incubated with the siRNAs for 48 h, the medium was changed, and 24 h later the cells were transfected a second time and incubated for an additional 24 h. Cell viability was assessed by trypan blue staining, and was equivalent in cultures treated with targeting and non-targeting siRNAs. Phagocytosis assays were performed 5 days after siRNA transfection.

### Phagocytosis Assays

Rod OS were isolated from bovine eyes [50] and covalently labeled with AlexaFluor 555 [52]. RPE-J cells were cultured for 6 days in eight-well chamber slides, and then incubated with 10 OS per cell for 4 h at 33°C. Unbound OS were removed by washing the cells 3 times with PBS containing 0.2 mM CaCl_2_ and 1 mM MgCl_2_, and the cells were fixed in 4% paraformaldehyde. To distinguish total and bound OS, duplicate samples were incubated before fixation with 0.2% trypan blue to quench fluorescence [53], as shown in Figure S2B. Slides were mounted using Prolong Gold containing DAPI, and were visualized by fluorescence microscopy (Eclipse E800; Nikon). Images were taken from 10–12 random fields (0.100 mm^2^ per field) for each group. OS with a diameter of at least 2 µm were quantified manually per viewing field. Counts of ingested AlexaFluor 555-labeled OS were obtained from trypan blue-treated samples, while total OS counts (bound plus ingested) were obtained from duplicate untreated samples. For each condition, assays were repeated three times and results represented as a mean ± standard error.

## Supporting Information

Figure S1 **Ion fragmentation patterns for MALDI-MS phosphopeptide analysis of rMERTK_571–864_.** Purified 6xHis-rMERTK_571–864_ was autophosphorylated by addition of ATP and digested by addition of porcine trypsin. Phosphopeptides were selected by TiO_2_ enrichment and separated by LC and subjected to MALDI-MS analysis. (A) Summary of peptides identified by MALDI-MS analysis. (B) Ion fragmentation data for fragments P1, P2, and P3 are shown. Peaks labeled with the letter “b” followed by numbers correspond to fragments with masses that have matches in the UniProt database. Autophosphorylation was detected at three sites in the catalytic domain of human MERTK (Y749, Y753, and Y754) in agreement with Ling et al. [19].
(TIF)

Figure S2 **Morphology of siRNA transfected RPE-J cells and quenching of AlexaFluor 555-OS fluorescence in RPE phagocytosis assays.** RPE-J cells were transfected with a pool of Grb2 targeting siRNAs or a non-targeting siRNA control. (A) The morphology of transfected cells was evaluated by immunostaining of ZO-1 and phalloidin staining of actin using fluorescence confocal microscopy. (B) Trypan blue quenching of the AlexaFluor 555-fluorescence on the surface of RPE-J cells transfected with a non-targeting siRNA that were fed OS fed for 2 hours, quenched, then fixed. Confocal images of representative fields show OS labeled in red, and DAPI-stained nuclei in blue.
(TIF)

Figure S3 **Rhodopsin colocalization with Pik3r1, Eea1, and Rab5 in OS fed RPE-J cells.** RPE-J cells were incubated with isolated bovine OS for 4 h. Cells were fixed and stained with anti-rhodopsin and anti-Eea1 (A), anti-Pik3r1 and anti-Eea1(B), or anti-Rab5 and anti-Eea1 (C), using AlexaFluor 488 and 555 secondary antibodies, with visualization using fluorescence confocal microscopy. Areas showing co-localization appear as yellow in the merged images and are marked by white arrowheads.
(TIF)

Table S1 **PCR primer sequences used to amplify transcripts encoding SH2-domain proteins.**
(DOCX)
