# Supplementary figures and images for: MERTK Interactions with SH2-Domain Proteins in the Retinal Pigment Epithelium
Source: PLoS One. 2013 Feb 4;8(2):e53964. doi: 10.1371/journal.pone.0053964 (PMC3563642; doi:10.1371/journal.pone.0053964)

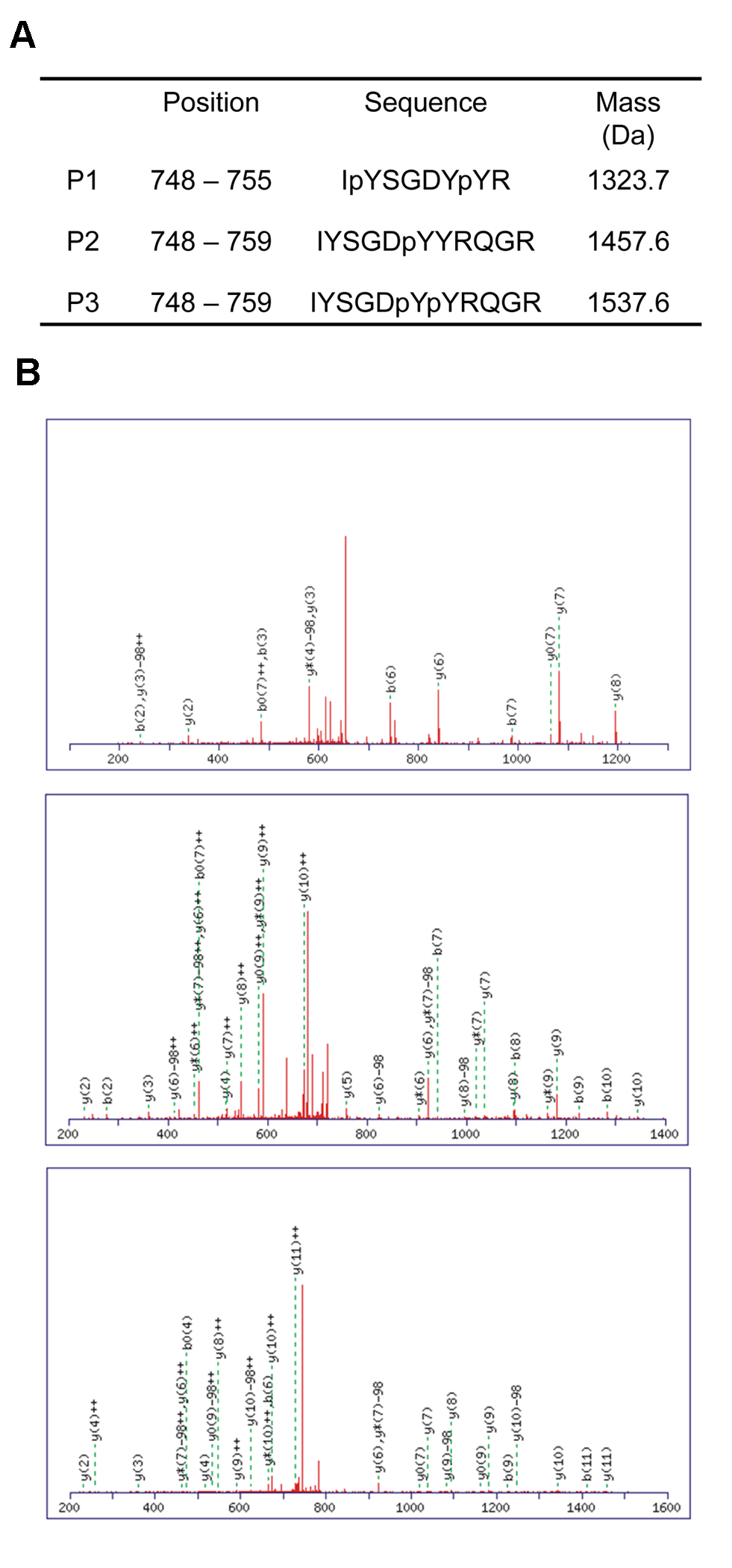

Supplement: Figure S1 — Ion fragmentation patterns for MALDI-MS phosphopeptide analysis of rMERTK571–864. Purified 6xHis-rMERTK571–864 was autophosphorylated by addition of ATP and digested by addition of porcine trypsin. Phosphopeptides were selected by TiO2 enrichment and separated by LC and subjected to MALDI-MS analysis. (A) Summary of peptides identified by MALDI-MS analysis. (B) Ion fragmentation data for fragments P1, P2, and P3 are shown. Peaks labeled with the letter “b” followed by numbers correspond to fragments with masses that have matches in the UniProt database. Autophosphorylation was detected at three sites in the catalytic domain of human MERTK (Y749, Y753, and Y754) in agreement with Ling et al. [19]. (TIF) [file pone.0053964.s001.tif]

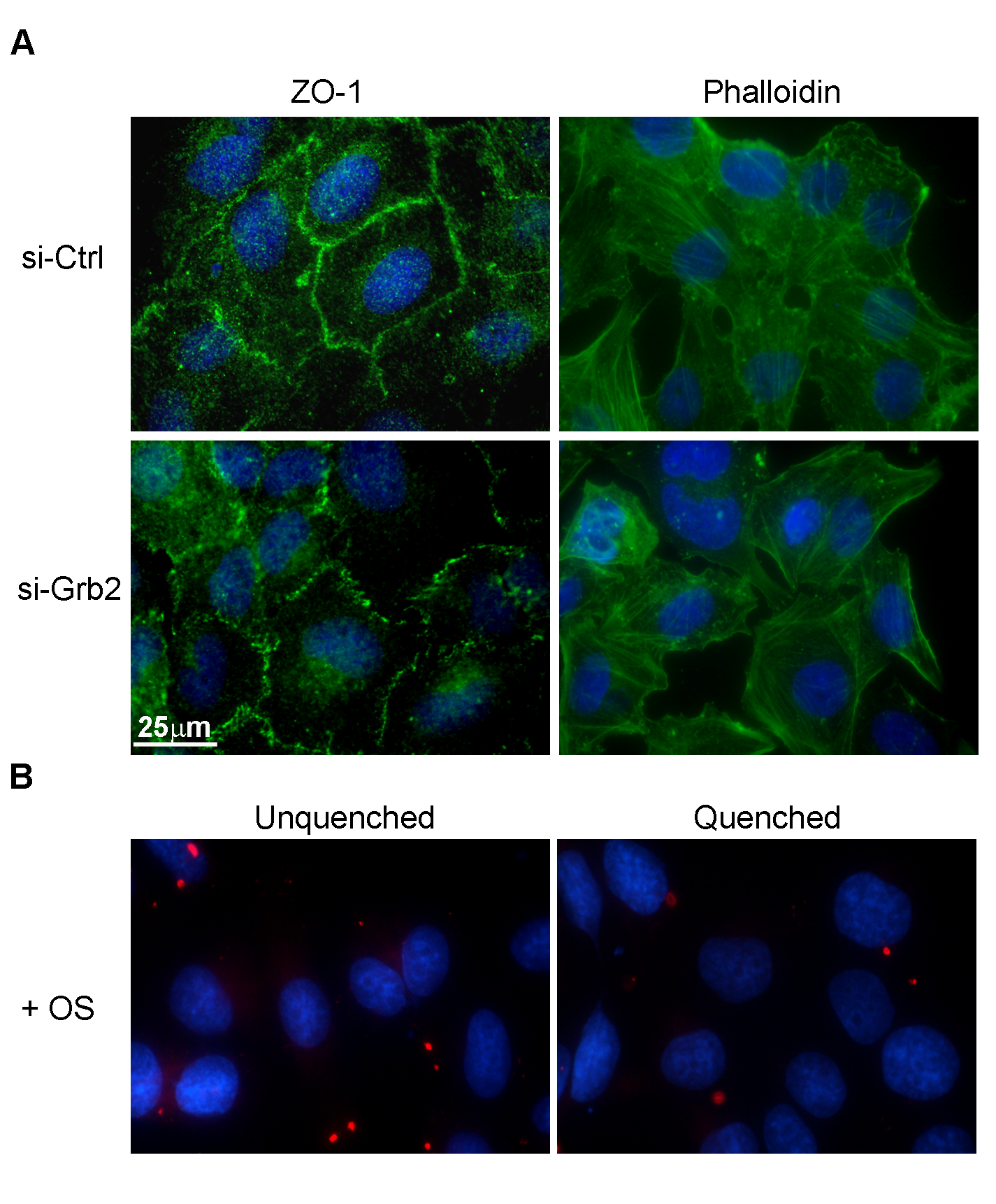

Supplement: Figure S2 — Morphology of siRNA transfected RPE-J cells and quenching of AlexaFluor 555-OS fluorescence in RPE phagocytosis assays. RPE-J cells were transfected with a pool of Grb2 targeting siRNAs or a non-targeting siRNA control. (A) The morphology of transfected cells was evaluated by immunostaining of ZO-1 and phalloidin staining of actin using fluorescence confocal microscopy. (B) Trypan blue quenching of the AlexaFluor 555-fluorescence on the surface of RPE-J cells transfected with a non-targeting siRNA that were fed OS fed for 2 hours, quenched, then fixed. Confocal images of representative fields show OS labeled in red, and DAPI-stained nuclei in blue. (TIF) [file pone.0053964.s002.tif]

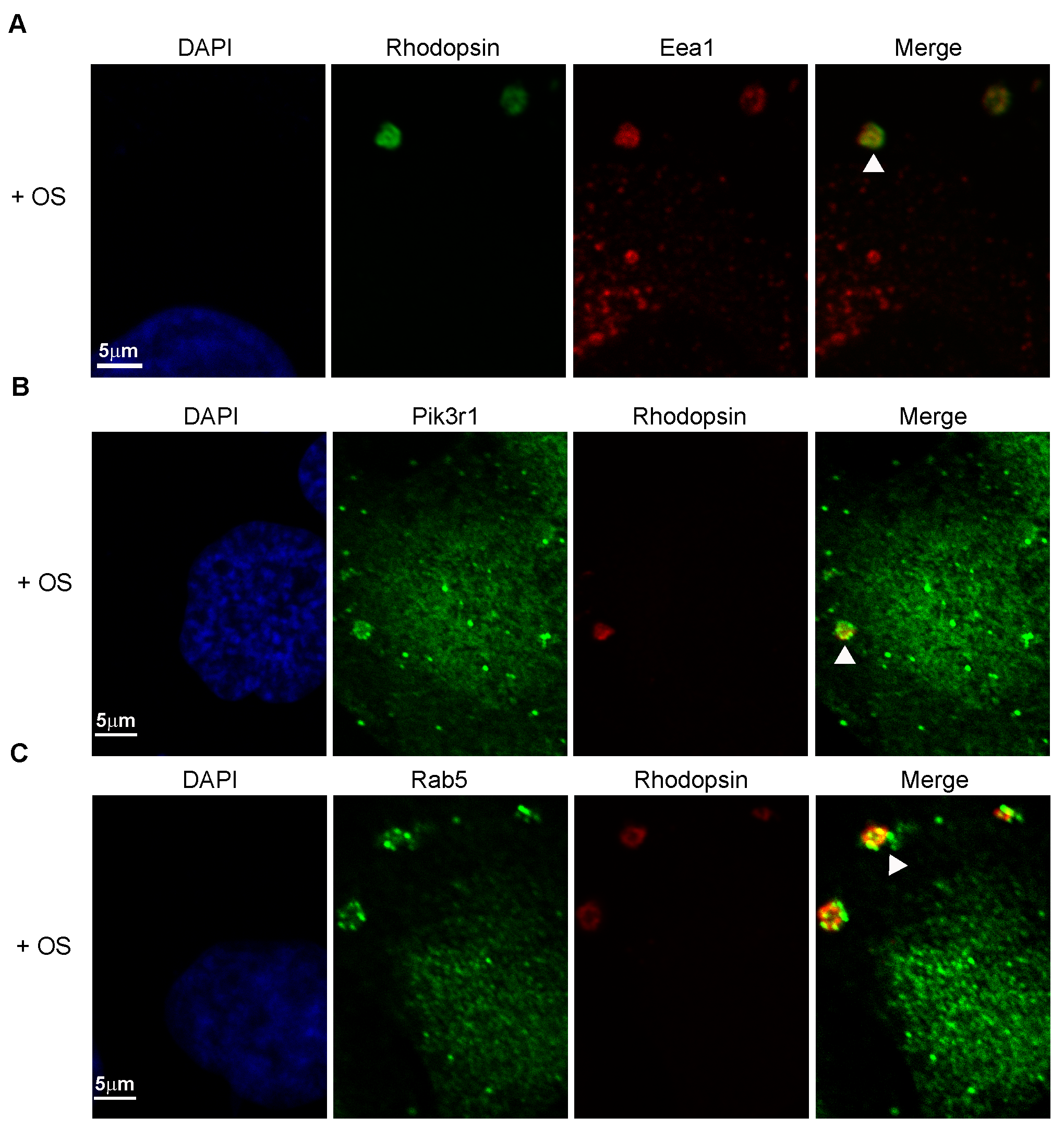

Supplement: Figure S3 — Rhodopsin colocalization with Pik3r1, Eea1, and Rab5 in OS fed RPE-J cells. RPE-J cells were incubated with isolated bovine OS for 4 h. Cells were fixed and stained with anti-rhodopsin and anti-Eea1 (A), anti-Pik3r1 and anti-Eea1(B), or anti-Rab5 and anti-Eea1 (C), using AlexaFluor 488 and 555 secondary antibodies, with visualization using fluorescence confocal microscopy. Areas showing co-localization appear as yellow in the merged images and are marked by white arrowheads. (TIF) [file pone.0053964.s003.tif]
