# Supplementary material for: MERTK Interactions with SH2-Domain Proteins in the Retinal Pigment Epithelium
Source: PLoS One. 2013 Feb 4;8(2):e53964. doi: 10.1371/journal.pone.0053964 (PMC3563642; doi:10.1371/journal.pone.0053964)
Supplement: Table S1 — PCR primer sequences used to amplify transcripts encoding SH2-domain proteins. (DOCX) [file pone.0053964.s004.docx]

**Supplementary Table 1.** PCR primer sequences used to amplify transcripts encoding SH2-domain proteins.

| **Gene** | **Forward primers 5’ to 3’** | **Reverse primers 3’ to 5’** |
| --- | --- | --- |
| Grb2 | GCCATCGCCAAATATGACTT | GAGCATTTCTTCTGCCTTGG |
| Grb7 | CAGCGCAGCCATTCATCGCA | TGGCTCTCCCGGACCAGGAAC |
| Grb10 | ATGCCTGGCGTAAGCGGAGC | AGGTCTGCCCATCATCCTCGCA |
| Pik3r1 | GTGGCTGGGGAATGAAAATA | CCGGTGGCAGTCTTGTTAAT |
| Vav1 | CTACGGGATCTGCTGATGGT | CTGCCGTAGGGTTTCATTGT |
| Vav2 | GCGAGAAGGTGATGTGGTGAAG | TAGGGCAGGTGATGGTGGAA |
| Vav3 | TTTCTGAGACGGATGGAAGG | ATGGAAGAGCCGAGTTGTCA |
| cSrc | TACACAGCCCGGCAAGGTGC | TCATGCAGGGACTCGGGGCA |
| Fgr | CGTGTGGTCCTTTGGGATTCTG | AGGCTGGTACTGTGGTTCTGTGGA |
| Fyn | ACACAGCAAGACAAGGTGCGAAGT | TCGTGCAGGGAGATCGGGCA |
| Yes | GCTGCTCAGATCGCTGATGGCA | GCCTCAGGAGCTGTCCACTTGA |
| Hck | CACCAAAGGGAGCTACTCGT | TCTTTCTCCCATGGCTTCTG |
| Lyn | TAGAAGAGCATGGGGAATGG | GAAAGCTCCTGCACTGTTCC |
| Lck | GGCAGCCCAGATTGCAGAGGG | GGGATTCGACCGTGGGTGACG |
| Hprt | CAAACTTTGCTTTCCCTGGT | CAAGGGCATATCCAACAACA |
